# Recent Progress in Mixed-Matrix Membranes for Hydrogen Separation

**DOI:** 10.3390/membranes11090666

**Published:** 2021-08-30

**Authors:** Chong Yang Chuah, Xu Jiang, Kunli Goh, Rong Wang

**Affiliations:** 1 Singapore Membrane Technology Centre, Nanyang Environment & Water Research Institute, Nanyang Technological University, Singapore 637141, Singapore; chongyang.chuah@ntu.edu.sg (C.Y.C.); xu.jiang@ntu.edu.sg (X.J.); gohkunli@ntu.edu.sg (K.G.); 2 School of Civil and Environmental Engineering, Nanyang Technological University, Singapore 639798, Singapore

**Keywords:** mixed-matrix membrane, zeolite, metal-organic framework, covalent organic framework, graphene, hydrogen separation

## Abstract

Membrane separation is a compelling technology for hydrogen separation. Among the different types of membranes used to date, the mixed-matrix membranes (MMMs) are one of the most widely used approaches for enhancing separation performances and surpassing the Robeson upper bound limits for polymeric membranes. In this review, we focus on the recent progress in MMMs for hydrogen separation. The discussion first starts with a background introduction of the current hydrogen generation technologies, followed by a comparison between the membrane technology and other hydrogen purification technologies. Thereafter, state-of-the-art MMMs, comprising emerging filler materials that include zeolites, metal-organic frameworks, covalent organic frameworks, and graphene-based materials, are highlighted. The binary filler strategy, which uses two filler materials to create synergistic enhancements in MMMs, is also described. A critical evaluation on the performances of the MMMs is then considered in context, before we conclude with our perspectives on how MMMs for hydrogen separation can advance moving forward.

## 1. Introduction

The global energy landscape is changing at a rapid pace, driven by the ever burgeoning world’s population and increasing demand from economic growth [1]. Today, the global energy outlook is also shaped by climate change and emerging opportunities from renewable energy [2,3]. Hydrogen (H_2_), at the crossroad between electricity and fuel, as well as long-term energy storage and potential low-carbon energy resource, is envisaged to play an important role in our efforts toward decarbonization and sustainability. However, being an energy carrier and not an energy source, hydrogen has to be first produced from primary energy sources that inevitably involve fossil fuel [4]. Hence, to enable hydrogen as a low-carbon energy resource, two approaches are at present widely undertaken and researched upon: (1) leveraging renewable electricity to drive an electrolysis process, which produces hydrogen (termed as green hydrogen) from water [5,6], and (2) cleaning the carbon emissions from hydrogen produced from natural gas to yield blue (decarbonized) hydrogen by means of carbon capture, storage and utilization (CCSU) [6,7,8]. As of 2020, green hydrogen is costly to produce, which is priced between USD 3.00–6.55 per kg hydrogen, due to the limited electrolysis capacity and the high cost of tapping on renewable energy [9]. Blue hydrogen, however, is relatively cheaper at USD 1.40–2.40 per kg hydrogen, but is heavily subjected to price fluctuation from the natural gas market, and the cost to implement CCSU technologies [10]. Notwithstanding both technological and cost constraints, the general consensus is that blue hydrogen will remain as the most attractive and viable option in the foreseeable future [11].

Membrane separation is a compelling technology for hydrogen purification and supporting CCSU efforts for blue hydrogen generation, owing to its cost-effectiveness, energy efficiency and modular design that favors easy retrofitting to existing plants [12,13,14,15]. The key driver of this technology is the gas separation membranes, which are currently well-represented by polymeric membranes and limited by a recurring permeability–selectivity tradeoff in performances [16,17,18]. Hence, in this review, we focus on one of the most extensively used and important strategies to overcoming this tradeoff, the mixed-matrix membrane (MMM) strategy. To begin the discussion, we first highlight the key technologies for today’s hydrogen generation. Then, under this background, we compile different separation technologies for hydrogen purification, comparing membrane technology to pressure-swing adsorption (PSA) and cryogenic distillation, as well as discussing their pros and cons. Thereafter, we summarize recent progress in MMMs (mostly in the last 10 years) for hydrogen separation, focusing especially on emerging filler materials, including zeolites, metal-organic frameworks (MOFs), covalent-organic frameworks (COFs) and graphene-based materials. Finally, we provide an overview of the binary filler strategy, which uses two filler materials to create synergistic enhancements, before giving a critical evaluation on the separation performances of MMMs and sharing our perspectives to help pave future efforts in this area of research.

## 2. Current Hydrogen Generation Market and Challenges

**Figure 1:**
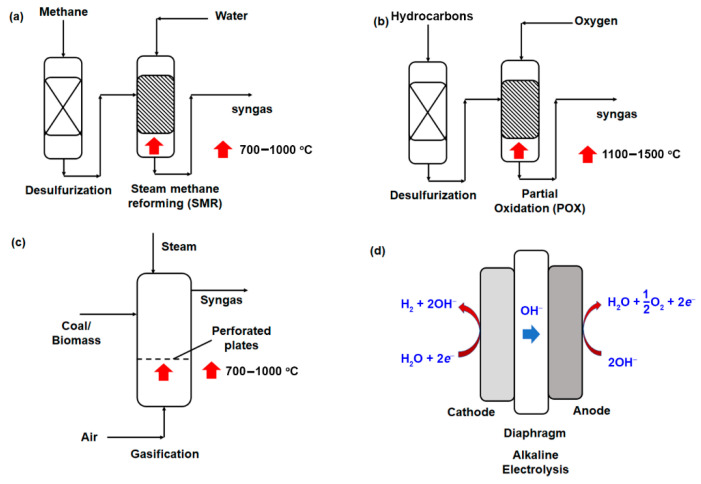
Methods to generate hydrogen gas from the feed (**a**) steam methane reforming (SMR), (**b**) partial oxidation (POX), (**c**) gasification, and (**d**) alkaline electrolysis. Reprinted with permission from Ref. [25], Creative Commons license CC BY 4.0.

Hydrogen is not an extractable resource, but created via synthetic means and separated from other elements before producing in its pure form [19]. Essentially, steam-methane reforming (SMR), partial oxidation (POX), gasification and electrolysis are currently the mainstream technologies available for hydrogen generation, with a market share of 48%, 30%, 4% and 18%, respectively, as cited in 2012 [20]. These technologies typically rely on the utilization of non-renewable fossil fuels (e.g., natural gas, coal and fuel oil) for hydrogen generation [21,22]. In 2018, it was reported that about 75% of the global hydrogen production (ca. 70 million tons) derived from the use of natural gas, which accounted for approximately 6% of the global natural gas supply [23,24]. In this section, a brief outline of each technology will be discussed (Figure 1).

First, SMR is a matured technology that utilizes high-temperature steam (ca. 700–1000 °C) to produce hydrogen from natural gas comprising mainly methane gas. The produced syngas has H_2_ and CO in the stoichiometric ratio of 3:1 (Equation (1)), which due to the endothermicity of the reaction, has resulted in the need for high operating temperature and steam-to-methane ratio of ca. 2.5–3.0 to minimize the formation of carbonaceous material (coke) [25]. CO molecules that are generated can be further converted to additional H_2_ by a water-gas shift (WGS) reaction as illustrated in Equation (2). Despite SMR being widely used today, the large CO_2_ emission, amounting to 830 million tons annually, is a major drawback in light of the current concern over global warming and climate change [19,23]. Thus, downstream carbon capture, sequestration and utilization are an important piece of the puzzle in realizing blue hydrogen production by SMR technology.

(1) $${\mathrm{CH}}_{4}+H_{2}O\to \mathrm{CO}+3H_{2},\;\Delta H_{298\;K}=206\;\mathrm{kJ}/\mathrm{mol}$$

(2) $$\mathrm{CO}+H_{2}O⇌{\mathrm{CO}}_{2}+H_{2},\;\Delta H_{298\;K}=-41\;\mathrm{kJ}/\mathrm{mol}$$

POX, however, creates syngas through the mixing of hydrocarbon fuel under sub-stoichiometric manner. As compared to SMR, this reaction occurs at a higher reaction temperature (1100–1500 °C) [26]. Considering that the reaction is exothermic, the reaction system can be built in a more compact manner, given that heat exchange is no longer required to maintain the operating temperature. The use of a catalyst is also an option to reduce the reaction temperature to between 600–900 °C and increase the hydrogen yield [26]. In recent years, there is increased use of oxygen enriched air to improve the heating value of the produced syngas [26]. Nevertheless, the syngas produced by POX has in general less hydrogen per unit of input fuel as compared to SMR, based on the stoichiometric chemical equation as provided in Equation (1) and (3) [27]. Besides, effective heat recovery is often challenging due to higher reaction temperature.

(3) $${\mathrm{CH}}_{4}+\frac{1}{2}O_{2}\to \mathrm{CO}+2H_{2},\;\Delta H_{298\;K}=-38\;\mathrm{kJ}/\mathrm{mol}$$

Gasification is another technology for the generation of syngas. It is an indirect combustion process at an elevated temperature to achieve complete combustion of raw materials at a sub-stoichiometric amount of oxidants [28]. Typically, solid fuels such as coal are used. Biomass is also gaining traction as a feedstock in recent years, owing to the acceleration of waste-to-energy initiatives that drive a circular economy. In comparison to the previous two approaches, the production of syngas via gasification requires the incorporation of both oxygen and steam as illustrated in Equation (3) and (4), respectively. Similar to POX, the heating value of the produced syngas from gasification can be enhanced by the use of oxygen-enriched air, although the overall energy efficiency is comparatively lower (35–50%) compared to POX system (70–80%) [20].

(4) $$C+\frac{1}{2}O_{2}\to \mathrm{CO},\;\Delta H_{298\;K}=-283\;\mathrm{kJ}/\mathrm{mol}$$

Last but not least, as discussed in the introduction, electrolysis is a process that uses electric current to split water molecules into hydrogen and oxygen molecules. Alkaline water electrolysis (AWE) is the most common approach to generate hydrogen, which involves the use of two electrodes–separated by a diaphragm to selectively allow hydroxide ion transport only in liquid alkaline electrolyte solution such as potassium hydroxide (KOH) or sodium hydroxide (NaOH) solution [29]. Among the four different technologies, AWE operates at the lowest operating temperature of between 30–80 °C with the concentration of the electrolyte typically in the range of 20–30% [30,31]. Recently, the use of proton exchange membrane (PEM) electrolysis in which the diaphragm used are typically composed of solid polysulfonated membrane (e.g., Nafion^®^, fumapen^®^) to allow proton (H^+^) transport, has been proposed as a viable alternative [32]. Notwithstanding the higher overall energy efficiency of 60–80%, electrolysis suffers from short technical lifetime (ca. 40,000 h) [20] and small production scale, which both hinder its adoption and eventual replacement of the conventional SMR as a mainstream hydrogen production technology. Besides, in the near future, the electricity used to power the electrolysis process will continue to derive from nonrenewable primary sources (e.g., fossil fuels), which results in not only inevitable negative environmental impacts, but also regressing to unsustainable practices.

## 3. Hydrogen Purification: Membrane vs. Other Technologies

**Figure 2:**
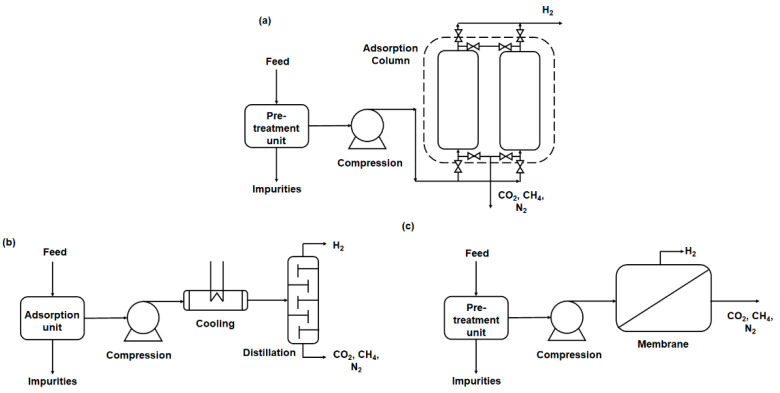
Illustration of (**a**) pressure swing adsorption (PSA), (**b**) cryogenic distillation, and (**c**) membrane-based separation. Reprinted with permission from ref. [34]. Copyright 2020 Elsevier.

All the aforementioned technologies do not produce pure hydrogen, which entails the need for purification to create hydrogen of targeted purity for its desired downstream applications. The common impurities associated with each hydrogen generation technology are summarized in Table 1. To date, pressure swing adsorption (PSA), cryogenic distillation and membrane-based separation are current technologies for hydrogen purification (Figure 2) in which key attributes will be elaborated in this section. We believe such discussions will be useful for readers to comprehend how membrane-based separation is different from the conventional PSA and cryogenic distillation.

PSA is the industrial standard for hydrogen purification. It has been reported that at least 85% of the current global hydrogen production units are dominated by the PSA technology [35] in which largescale production capacity up to 400,000 Nm^3^/h has been successfully developed by the Linde group [36]. PSA involves the utilization of micro- and mesoporous solid adsorbents (e.g., zeolites, activated carbons, alumina and silica gels) that are packed in an adsorption column for separation. Due to the small polarizability and quadrupole moment of hydrogen gas in comparison to other impurities such as carbon dioxide (CO_2_), nitrogen (N_2_) and methane (CH_4_), a pressurized gas feed up to 40 bar is necessary to induce effective adsorption of polarizable gas impurities by the adsorbents [19,37]. High-pressure hydrogen, which cannot be adsorbed, will be recovered at the top of the adsorption columns. In general, PSA possesses the capability to achieve high hydrogen purity (99.999%) [38], albeit a typical 65–90% hydrogen recovery [16]. Besides, PSA operates on a cyclic basis to regenerate spent adsorbents for subsequent adsorption process. Thus, PSA operation is comparatively economical if high input gas and high flow rate are utilized in the process [35,38].

Conversely, cryogenic distillation purifies based on boiling point differences. Hydrogen has the lowest boiling point (−252 °C at 1 bar pressure), and thus the most undesirable impurities can be condensed into a liquid phase and separated from the targeted hydrogen [39]. Cryogenic distillation offers higher handling capacity with higher hydrogen recovery, but at the same time, higher cost incurs from gas compression at cold operating condition, and lower purity level of the extracted hydrogen than PSA, with the highest purity reported at 99% [38]. Hence, a hybrid system combining PSA and cryogenic distillation is proposed in which the waste gas from the PSA operation (with high CO_2_ content) is sent to cryogenic distillation for further hydrogen extraction [40]. Such an approach is currently adopted by Airliquide under the tradename CryoCap^TM^ H_2_ technology [41]. As cryogenic distillation operates at a low temperature, purified hydrogen can be readily stored in liquid phase, allowing for a more efficient transportation as compared to pressurized hydrogen gas. Cryogenic distillation, similar to PSA, is competitive only at large-scale operation and when the feed gas hydrogen concentration is low [34].

As compared to PSA and cryogenic distillation, hydrogen purification by membrane-based separation is competitive in its own right and is considered a potential tool to generate hydrogen-enriched gas stream [42,43]. Membrane separates via a difference in the gas permeation rates between hydrogen and other impurities, which is dictated mainly by a solution-diffusion mechanism [16,17]. In the solution-diffusion mechanism, the permeability of a gas is a product of its diffusivity and solubility [44]. Hydrogen has a higher diffusivity, as it can diffuse faster than other gas constituents, considering that hydrogen molecules possess a small kinetic diameter [45]. Nonetheless, due to its small polarizability, the solubility of hydrogen in a membrane is considerably lower than other polarizable gases such as CO_2_ [19]. Thus, understanding the mass transport mechanism is critical to the success of membrane-based hydrogen separation. Unlike PSA and cryogenic distillation, membrane-based separation can be more energy-efficient and cost-effective. This is attributed to the feasibility of membranes to perform gas separation without a phase change or desorption process, which is needed in cryogenic distillation and PSA [46,47]. In addition, it requires lower capital investments due to ease of retrofitting and smaller footprint. Thus, membrane-based separation is able to value-add in areas not possible by the other two technologies. However, the membrane technology is also limited to a smaller feed stream flow rate, moderate hydrogen purity output (90–95%) and lower overall recovery rate (85–90%) [48].

**Figure 3:**
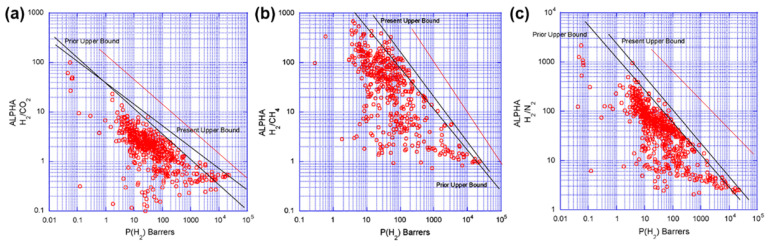
Robeson plot showing the present (2008) and prior (1991) upper bound for (**a**) H_2_/CO_2_, (**b**) H_2_/CH_4_, and (**c**) H_2_/N_2_ gas pairs. Additional line in brown represents the most well-received upper bounds based on up-to-date profiles obtained from the following references [56,57,58]. Reprinted with permission from ref. [17]. Copyright 2008 Elsevier.

At present, polymeric membranes have a dominant presence in the membrane market for hydrogen separation, owing to the suitability and economical large-scale processing of polymeric materials into separation membranes. However, given the solution–diffusion mechanism as driven by their dense membrane structures, polymeric membranes continue to be challenged by the permeability–selectivity tradeoff in all industrially relevant gas pairs (H_2_/CO_2_, H_2_/CH_4_ and H_2_/N_2_, see Figure 3). Hence, one primary focus is on resolving this tradeoff issue in hydrogen separation membranes. Among the many different types of membranes, including (nano)laminated and thin-film (nano)composite membranes that are studied to date [49,50,51], MMMs are one of the most well-researched and promising approaches [52,53]. Defined as the incorporation of filler materials into continuous polymer matrices, MMMs have the clear objective of capitalizing filler materials to tune the transport properties of the polymer matrices. In this regard, filler materials, such as zeolites, MOFs, COFs, and graphene-based materials, will be covered in this review. While there are other filler materials such as carbon nanotubes, which have been adopted for hydrogen separation [54,55], these filler materials are emerging and gaining importance in recent years. Binary filler strategy using these fillers is also at times prepared to create synergistic performance enhancements. For these reasons, in the following section, we delve into this exciting field of research, discussing current state-of-the-art MMMs that contain these filler materials, their merits and limitations, as well as effects on the membrane performances for hydrogen separation.

## 4. Mixed-Matrix Membranes for Hydrogen Separation

### 4.1. Zeolite-Based Membranes

Zeolites are microporous crystalline aluminosilicates, which are interconnected via SiO_4_ and AlO_4_ tetrahedron primary units. As a group of inorganic microporous materials, zeolites have been widely studied for gas storage and separation due to their large specific area and excellent physicochemical stability [59,60,61,62,63]. Zeolites can be synthesized in different shapes, particle sizes, and pore diameters for various gas separation applications. For H_2_ separation, MMMs can either be H_2_-selective or reverse-selective, depending on the polymer and zeolite selection [64]. For example, MFI, SAPO-34, and SSZ-13 are CO_2_-selective zeolites, owing to the CO_2_ favored competitive adsorption [64,65,66]. Hu et al. reported a MMM consisted of Pebax^®^1657, ionic liquid (IL), and surface modified SAPO-34 [67]. The SAPO-34 was decorated with –NH_2_ groups to enhance its interfacial compatibility with the polymer matrix. The presence of IL elevates the CO_2_ affinity, resulting in a high CO_2_/H_2_ selectivity of 22.1 (Table 2) [67].

Most of the zeolites are adopted for H_2_-selective membranes due to molecular sieving effect by their narrow pores. For example, in Matrimid^®^ 5218/DDR zeolite MMMs, the appropriate pore size of DDR zeolite (3.6 × 4.4 Å) played a crucial role in achieving a high H_2_/CH_4_ selectivity of 129.8 since the pore diameter falls in between the diameter of H_2_ (2.9 Å) and CH_4_ (3.8 Å) [68]. Similarly, zeolite 4A with pore diameter of 3.8 Å was also found to be able to significantly enhance the H_2_/N_2_ and H_2_/CH_4_ selectivity of its MMMs [69,70]. To overcome the interfacial compatibility issue, Esmaeili et al. adopted nanosizing and silanization strategies to increase interfacial interaction between zeolite 4A and polyvinyl acetate (PVAc), as to reduce zeolite 4A agglomeration in MMMs, leading to better filler dispersion and increase in H_2_ selectivity [71]. A recent study by Eden et al. also saw the use of hydroxyl sodalite with a cage structure and small pore diameter of 2.6 Å [72]. By infusing 10 wt.% of hydroxy sodalite filler in PSF matrix, a huge increase in H_2_ permeance from 2.7 × 10^−9^ mol m^−2^ s^−1^ Pa^−1^ to 7.3 × 10^−9^ mol m^−2^ s^−1^ Pa^−1^ was observed under single gas evaluation (Table 2). This was accompanied by a superior H_2_/CO_2_ selectivity of 54.9 when tested under mixed-gas evaluation [72].

**Figure 4:**
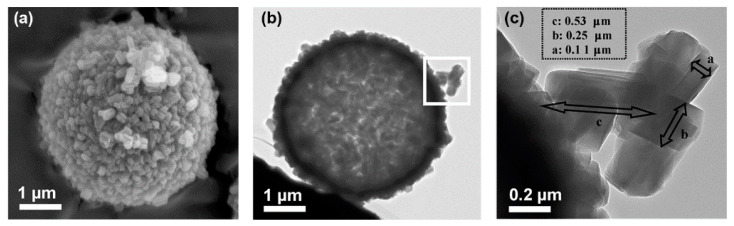
Structural morphology of HZSs, showing the (**a**) scanning electron microscopic (SEM), (**b**) transmission electron microscopic (TEM) images, and (**c**) the silicalite-1 crystals on the shell of the hollow sphere. Reprinted with permission from ref. [77]. Copyright 2011 Elsevier.

On a different note, spherical particles of ordered mesoporous silica have strong capacity to minimize filler agglomeration when used in MMMs, owing to the spherical shape and lower surface area to volume ratio [73,74,75]. For example, Tseng et al. reported a series of SBA-15-derived MMMs containing SBA-15 particles of different shapes and sizes and found that a spherical shape with particle diameter of 1.6 μm had the best compatibility with the polymer matrix [76]. To reduce the pore diameter of the mesoporous silica to a range suitable for H_2_ separation, Zornoza et al. revealed a layer-by-layer (LBL) procedure to synthesize silicalite-1 crystals over the spherical silica, giving rise to hollow zeolite spheres (HZSs) (Figure 4) [77,78]. The HZSs were used as filler materials in polysulfone (PSF) and Matrimid^®^ 5218 and 6FDA-DAM matrices. At 8 wt.% loading, H_2_ permeability could reach 15, 38 and 541 barrer, with H_2_/CH_4_ selectivity of 80, 180 and 25, respectively (Table 2) [77,78]. The enhanced H_2_ permeability was attributed to the hollow spheres, giving rise to low transport resistance and higher free volume as a result of the disruption to the polymer chains. More importantly, the silicalite-1 shell offered a regular pore diameter of ~5.5 Å and increased surface roughness of the hollow spheres, leading to improved polymer-filler interfacial interaction and enhanced H_2_/CH_4_ selectivity.

Generally, the pore size of most zeolites is too large for a highly efficient H_2_ molecular sieving from other gases, especially for H_2_/CO_2_ separation. For instance, well-known zeolitic structures, such as MFI, CHA, LTA and FAU (notation defined based on International Zeolite Association, IZA), that are commonly used for post-combustion CO_2_ capture possess pore size of 3.8 Å, 3.8 Å, 4.8 Å and 7.4 Å, respectively, which are considerably larger with respect to the kinetic diameter of H_2_ molecules (2.9 Å) [44]. Hence, there is limited research focusing on zeolite-based MMMs for H_2_ separation. However, in recent years, emerging advanced nanomaterials, such as MOFs and COFs with narrower and more orderly channels as well as diverse chemical properties, have become a more popular choice for achieving high-performance MMMs for H_2_ separation.

### 4.2. MOF-Based Membranes

Compared to the pure inorganic zeolites, MOFs are a group of porous organic-inorganic hybrid materials consisting of metal ions/clusters and rigid organic ligands [79]. The ultrahigh specific areas, adjustable micropore structures, and easily modified surface chemical properties endow MOFs with excellent gas adsorption and molecular sieving abilities, which are attractive for gas separation membranes in recent decades [80,81].

Similar to any other MMMs, the good interfacial compatibility of MOF nanoparticles and polymer matrix is the key prerequisite for better gas separation performance [82,83]. By utilizing functionalized ligands, MOFs could be easily decorated with functionalities such as –OH, –NH_2_, and –NO_3_ via bottom-up approaches, thereby improving their compatibility with the organic polymers. Cao et al. adopted NH_2_-functionalized CAU-1-NH_2_ as the filler for poly(methyl methacrylate) (PMMA) membranes [84]. The hydrogen bonds between CAU-1-NH_2_ and PMMA led to a compact and stable MMMs with an ultrahigh H_2_ permeability (>1 × 10^4^ barrer) and a good H_2_/CO_2_ selectivity (>10) (Table 3) [84]. Hu et al. reported a high selective UiO-66(Hf)-(OH)_2_/polybenzimidazole (PBI) MMM with an excellent H_2_/CO_2_ selectivity of 19.4 [82]. Ma et al. also used UiO-66-(OH)_2_ as filler for a PI matrix. The obtained UiO-66-(OH)_2_/PI MMMs exhibited a H_2_ permeability of up to 907 barrer and a H_2_/CH_4_ selectivity up to 42 [85]. Apart from these examples, several other functionalized MOFs, such as MIL-53-NH_2_ [86,87] and UiO-66-NH_2_ [88,89], were also demonstrated.

Post-synthetic modification is another promising approach to modifying MOF particles toward better separation performances [90,91,92,93,94]. Al-Maythalony et al. performed a linker exchange of benzimidazolate to benzotriazolate for ZIF-7 nanoparticles to tune the performance of ZIF-7/poly(ether imide) (PEI) membranes [95]. The resulting membranes with post-synthetically modified ZIF-7 filler displayed the highest gas permeability for all test gases with a H_2_ permeability of 2021 barrer. Sánchez-Laínez et al. also obtained ZIF-93/11 hybrid nanoparticles by immersing synthesized ZIF-93 particles into benzimidazole solution [96]. The hybrid ZIF-93/11 particles were then applied as a filler for PBI-based MMMs, leading to H_2_ permeability of 207 barrer and H_2_/CO_2_ selectivity of 7.7 at a high operating temperature of 180 °C.

**Figure 5:**
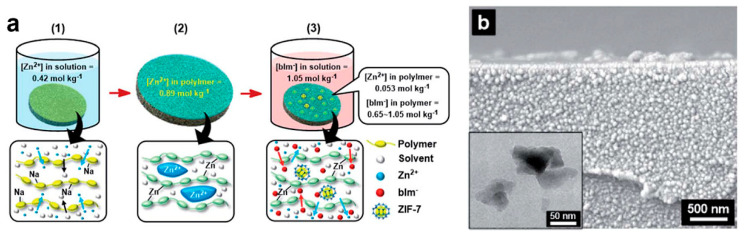
(**a**) Illustration of the in situ growth of ZIF-7 in PI matrix. (**b**) Scanning electron microscope image of the cross-section of the ZIF-7/PI MMM. The inset shows a transmission electron microscope image of the in situ formed phase III ZIF-7 fillers in the PI matrix. Reprinted with permission from ref. [99]. Copyright 2020 Royal Society of Chemistry.

Alternative to modifying the filler materials, post-synthetic engineering of the membrane formation process can also help to resolve the polymer-filler interfacial challenge. Kim et al. found that post-synthetic thermal-rearrange (TR) could effectively alleviate the interfacial voids between ZIF-8 nanoparticles and TR-polymers [97]. With a 90% TR conversion and 20 wt.% ZIF-8 loading, the MMM reached a H_2_ permeability of 1206 barrer with a H_2_/N_2_ selectivity of 21.3 (Table 3). Furthermore, Xiang et al. developed a H-bonding assisted LbL assembling strategy to synthesis MMMs, containing phenyl acetyl functionalized MOFs with poly(acrylic acid) (PAA) [98]. Owing to the strong filler-matrix interaction, the resulting MMMs exhibited a high H_2_ to CO_2_ selectivity of 20.3 (Table 3). As opposed to conventional blending-based MMM fabrication, Park et al. applied polymer-modification-enabled in situ MOF formation (PMMOF) method to synthesize ZIF-7/PI MMMs [99]. The crystal phase of the ZIF-7 filler was tunable within the PI matrix, giving a phase III ZIF-7-based MMM with H_2_ permeability of 1630 barrer and H_2_/CO_2_ selectivity of 3.8 (Figure 5).

To further enhance the polymer-filler interfacial compatibility, polydopamine can be used as a compatibilizing layer [100,101], which is typically coated onto MOF particles either by post-synthesis treatment or in situ polymerization during MOF synthesis. Wang et al. synthesized ZIF-8-PD by dispersing synthesized ZIF-8 nanocrystals into polydopamine precursor solution [102]. The coated ZIF-8-PD particles showed great compatibility with PI matrix, resulting in significant enhancement in gas permeability at almost no compromise in gas selectivity [102]. In comparison, Jiang et al. reported an in situ dopamine-modulated synthesis method to obtain monodispersed ZIF-8-DA particles in one step [103]. The incorporation of monodispersed ZIF-8-DA particles in Matrimid^®^ 5218 membranes was able to demonstrate simultaneously improved H_2_ permeability and H_2_/CO_2_ selectivity.

**Figure 6:**
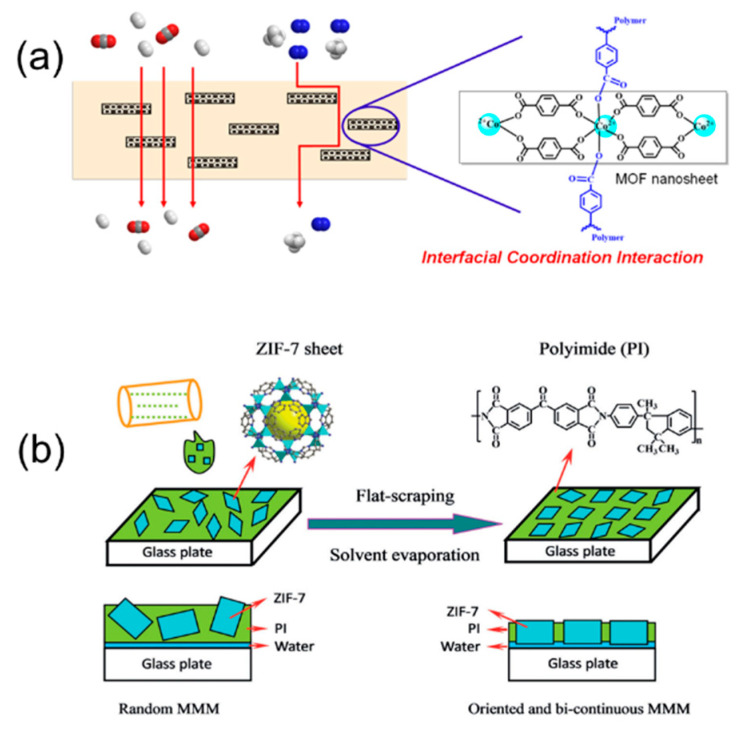
Illustration of (**a**) the 6FDA-Durene-DABA/CBMN MMM. Reprinted with permission from Ref. [113], Copyright 2020 American Chemical Society, and (**b**) a schematic showing the preparation of the oriented and penetrating ZIF-7@PI MMM. Reprinted with permission from ref. [85]. Copyright 2019 Wiley-VCH.

Compared to MOF nanoparticles, two-dimensional (2D) MOF nanosheets are more efficient fillers for MMMs since the partially oriented nanosheets with high-aspect-ratio will increase the chance of sieving the gas molecules [104,105,106,107,108]. ZIF-L is one of the most accessible 2D MOFs [109,110]. Kim et al. reported that the MMM containing 20 wt.% dopamine modified ZIF-L could get a 550% improvement in H_2_/CO_2_ selectivity (from 1.7 to 13.4) (Table 3) [111]. Deng et al. synthesized two different types of ZIF-L with the selection of different metal sources, and revealed that ZIF-L-Co was better than ZIF-L-Zn in elevating the H_2_ permeability of MMMs [112]. MMM with 20% ZIF-L-Co showed the best H_2_ permeability at 1986 barrer. Bi et al. also incorporated ultrathin Co-benzenedicarboxylate MOF nanosheets (CBMNs) into a 6FDA-Durene-DABA matrix (Figure 6a), leading to improved H_2_ separation with a H_2_/CH_4_ selectivity of up to 42 [113]. Recently, Ma et al. proposed a new concept of fabricating MMMs [85]. Instead of embedding the filler in the polymer matrix, the ZIF-7 nanosheets were oriented and penetrated through the PI matrix, given that the thickness of the ZIF-7 nanosheets was larger than the PI membrane (Figure 6b). Through this design, the channels of the penetrating ZIF-7 nanosheets served as the dominant pathways for gas molecules, resulting in extremely high molecular sieving performance observed for H_2_ separation. The H_2_/CO_2_ and H_2_/CH_4_ selectivity could reach 91.5 and 128.4, respectively, with a H_2_ permeance of 3.0 × 10^−7^ mol m^−2^ s^−1^ Pa^−1^. Despite the high promise of using MOF nanosheets in MMMs for H_2_ separation, one of the key challenges lies in the production of high-quality MOF nanosheets to support the large-scale membrane fabrication [114].

### 4.3. COF-Based Membranes

COFs are a class of microporous organic polymers—joining porous aromatic frameworks (PAFs), conjugated microporous polymers (CMPs), and hyper-crosslinked polymers (HCPs) etc.—composed of light elements connected via covalent bonds [115,116,117]. Contrary to inorganic zeolites and organic-inorganic hybrid MOFs, COFs are purely organic, and have the natural advantage of good interfacial compatibility with organic polymers [118]. Moreover, the covalently bonded backbones of COFs endow these materials with a better chemical stability than MOFs, making them better filler materials for gas separation under harsh conditions. Similar to zeolites, the pore sizes of most COFs are too large to show any discriminatory sieving of H_2_ from other gases. Therefore, nanolaminated COF membranes are always composited with other materials such as MOFs and GO to form dual-layer/composite membranes [119,120,121,122]. Another way to effectuate precise molecular sieving is to fabricate vertically aligned COF membranes to allow gas molecules to separate via the narrow nanochannels defined by the interlayer spacing between COF nanosheets [123]. However, due to the high cost and technical challenges in fabricating these types of membranes, MMMs are still the preferential membrane type for COF-based membranes.

As an emerging filler material, COFs are frequently adopted to enhance the poor selectivity and physical aging resistance of highly permeable glassy polymers [124]. For example, the size-controlled HCPs were used to redeem the gas selectivity and the long-term stability of the ultra-permeable poly(1-trimethylsilyl1-propyne) (PTMSP) membrane as reported by Hou et al. [125]. Owing to the sufficient HCP-PTMSP interaction and efficient dispersion of the HCP particles, H_2_/CH_4_ and H_2_/N_2_ selectivity were enhanced by 690% and 540%, reaching up to 22 and 30, respectively. The same group also added PAF-1 additives into the highly permeable TPIM-2 polymer matrix, leading to simultaneous improvement in the H_2_ permeability, H_2_/N_2_ selectivity, and long-term stability of the MMM [126]. PAF-1 was also used to elevate the H_2_ separation performance of PIM-1 membranes. The addition of PAF-1 filler in PIM-1 not only improves the H_2_ permeability of the membrane to 5500 barrer (Table 4), but more importantly, also endows the MMM with a good physical aging resistance as exemplified by a higher H_2_/N_2_ selectivity with age [127].

**Figure 7:**
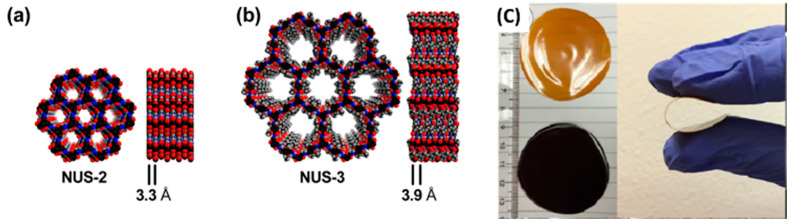
Crystal structures, showing a pore size of (**a**) NUS-2 as compared to (**b**) NUS-3, and (**c**) photo images of 20 wt %NUS-2@Ultem (brown) and 20 wt %NUS-3@Ultem (black). Reprinted with permission from ref. [130]. Copyright 2016 American Chemical Society.

Similar to 2D MOFs, 2D COFs are equally attractive for MMMs. Many COFs have 2D laminar structures that can be easily exfoliated into high-aspect-ratio nanosheets for improving membrane selectivity [128,129]. For example, the incorporation of a type of 2D COF, named NUS-2 (Figure 7), at 20 wt.% loading in PBI matrix showed an improved H_2_/CO_2_ selectivity from 9.5 to 31.4 with a slight improvement in H_2_ permeability (Table 4) [130].

**Figure 8:**
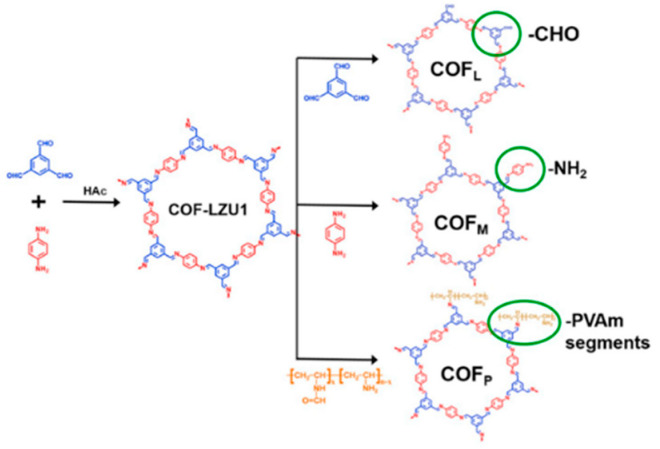
Synthesis process of functionalized COF_L_, COF_M_ and COF_P_. Reprinted with permission from ref. [131]. Copyright 2020 Elsevier.

Furthermore, given the rich chemistry and functional groups, the organic structure of COFs is an ideal platform to realize molecular level fusion with polymer matrix. Cao et al. grafted chemical functional groups, such as –CHO and –NH_2_, and polymer segments of poly(vinyl amine) (PVAm) onto a 2D COF (COF-LZU1) before blending with the PVAm matrix (Figure 8) [131]. The results indicated that the MMMs incorporated with PVAm grafted COFs showed a reverse selectivity with CO_2_/H_2_ selectivity highest at 22.2. Recently, Huang et al. developed an in situ generation strategy to synthesis polymer molecular sieve (PMS) within the polymer matrix, which can load as high as 70 wt.% PMS in the MMMs [132]. The obtained MMMs exhibited a high H_2_/CH_4_ selectivity of 183.

### 4.4. Graphene-Based Membranes

**Figure 9:**
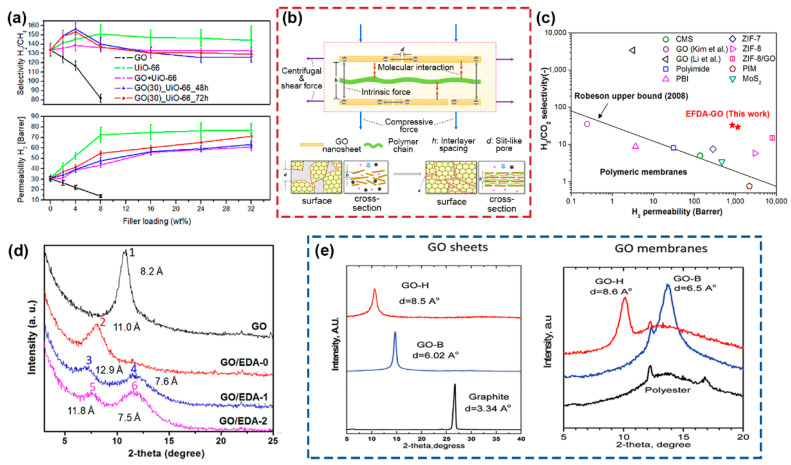
(**a**) Performance of mixed-matrix membranes containing graphene oxide (GO), showing GO, UiO-66 and UiO-66/GO effect on H_2_ permeability and H_2_/CH_4_ selectivity in Matrimid^®^ 5218 polymer matrix. Reprinted with permission from ref. [135]. Copyright 2017 Elsevier. (**b**) An illustration of the GO-laminated membrane intercalated by polyethyleneimine and (**c**) its H_2_/CO_2_ performance with reference to the 2008 Robeson upper bound. Reprinted with permission from ref. [139]. Copyright 2016 American Chemical Society. (**d**) Comparison of the XRD profile and *d*-spacing between GO membranes and GO membranes intercalated with ethylenediamine (EDA). Reprinted with permission from ref. [140]. Copyright 2018 Elsevier. (**e**) Effects of two different GO synthesis methods (GO-H, GO synthesized by modified Hummer’s method and GO-B, GO synthesized by modified Brodie’s method) on the *d*-spacing of the GO sheets and laminated membranes. Reprinted with permission from ref. [141]. Copyright 2019 Royal Society of Chemistry.

Graphene consists of a single-layer *sp^2^*-hybridized carbon atoms arranged in a hexagonal honey-comb lattice [133,134]. The applicability of graphene-based materials, especially GO, in membranes for gas separation has been an active area of research, owing to its 2D morphology and monoatomic thickness, which theoretically gives the lowest possible transport resistance. Castarlenas et al. demonstrated the potential utility of GO in PSF and Matrimid^®^ 5218 polymer matrices for H_2_ separation [135] in which MMMs showed undesirable decrease in both H_2_/CH_4_ selectivity (~50% and 38%, respectively) and H_2_ permeability (~140% and 100%, respectively) with increased GO loading from 4 wt% to 8 wt% (Figure 9a and Table 5). The decrease in H_2_ permeability stemmed from the nonporous nature of GO that resulted in gas diffusion taking up an extensively tortuous pathway across the membranes. Considering that CH_4_ possesses a higher polarizability than H_2_ [45], the decrease in H_2_/CH_4_ selectivity may be attributed to the favorable interaction between CH_4_ and the functional groups on the GO. This is evidenced by the contradictory increase in mixed-gas CO_2_/CH_4_ selectivity with increase in GO loading, suggesting the presence of strong GO interaction with the gas molecules [136,137]. Albeit the drop in H_2_ permeability with GO loading, graphene-based materials show a strong capacity for improving the mechanical properties of MMMs. For instance, 10 wt% loading of GO nanosheets in Matrimid^®^ 5218 matrix showcased more than 100% increment in the Young’s modulus of the membranes [138].

On this account, GO can be designed as ultrathin laminates on top of polymeric membrane substrates to avoid excessive compromise in the membrane’s gas permeability. GO-laminated membranes conduct molecular sieving by using the interlayer spacing between GO nanosheets that serves as well-defined nanochannel for molecular transport. Precise control of the size of the interlayer spacing is key to achieving selective gas transport and separation, and this feat can be realized by intercalating different spacers between GO nanosheets [133,142]. While laminated membranes deviate from the usual MMMs in which filler materials are mixed within dense matrices, the design and outcome are similar when polymeric spacers are used. Hence, we report only GO-laminated membranes that are intercalated by polymers to align with the scope of this review. Shen et al. assembled GO with 0.1 wt.% polyethyleneimine to fine-tune the interlayer spacing to within 0.4 nm [139]. By applying an external force driven assembly (EFDA) method comprising a vacuum-spin technique, they were able to obtain a highly ordered laminar structure (Figure 9b) that exhibited H_2_ permeability of between 840–1200 barrer and H_2_/CO_2_ selectivity of between 29–33, surpassing the 2008 Robeson upper bound (Figure 9c and Table 5).

Another study conducted by Lin et al. [140] utilized ethylenediamine (EDA) as the polymer spacer to fabricate GO-laminated membranes, given that EDA can be cross-linked to the –COOH groups on GO nanosheets. The cross-linking time between GO and EDA (GO/EDA-X where X = 0, 1 and 2 in hours) was varied, and two different stacking patterns were observed with the increase in cross-linking time (i.e., GO/EDA-1: *d*-spacing = 12.9 Å and 7.6 Å; GO/EDA-2: *d*-spacing = 11.8 Å and 7.5 Å), as opposed to GO/EDA-0, which possessed a single *d-*spacing of 11.0 Å (Figure 9d). The two types of stacking arose from: (1) an increase in the interlayer spacing due to the presence of EDA molecules, and (2) a decrease in interlayer spacing due to GO reduction by the EDA. Owing to these changes, a sharp decrease in H_2_ permeance and an increase in H_2_/CO_2_ selectivity were reported as compared to pure GO membrane. A similar investigation was also conducted by Cheng et al. [143] in which cysteamine, which contains both amino and thiol functional groups, cross-linked with GO nanosheets to give a smaller interlayer spacing that led to an enhancement in H_2_/CO_2_ selectivity, reaching above 20 (Table 5).

On a different note, the interlayer spacing of GO nanosheets can also be tuned with the use of different synthesis methods. In general, the most common approach in GO synthesis is via a modified Hummers’ method (GO-H), considering that this approach allows rapid graphite oxidation [144]. However, it is generally difficult for GO membranes made from this GO-H to possess an interlayer spacing that is smaller than 8 Å. As a demonstration, in the study conducted by Ibrahim et al. [141], GO membranes was assembled from GO synthesized using a modified Brodie’s method (GO-B) [145]. GO-B gave GO-laminated membranes with smaller interlayer spacing (Figure 9e), and this resulted in a substantial increase in H_2_/CO_2_, H_2_/N_2_ and H_2_/CH_4_ selectivity of up to 129%, 91% and 76%, respectively, as compared to GO-H. However, GO synthesis via the modified Brodie’s method is comparatively more complicated than the modified Hummer’s method [144], as successive graphite oxidation is necessary to achieve the desired oxidation (i.e., C/O ratio > 2), and this added complexity should be considered for graphene-based membrane fabrication.

### 4.5. Binary Fillers

As discussed in Section 4.4, using GO alone as a filler for MMMs is less effective due to its undesirable decrease in H_2_ permeability. Thus, the use of binary fillers, which includes incorporating two separate fillers into the polymer matrix or combining two fillers into three-dimensional (3D) composites, has been demonstrated to be promising for enhancing H_2_ separation performances [138,146]. For example, Castarlenas et al. [135] explored MMMs using two different types of filler: (1) a UiO-66/GO composite, and (2) individual UiO-66 and GO fillers physically blended into the polymer matrix. Enhancements in both H_2_ permeability and H_2_/CH_4_ selectivity were observed when using UiO-66/GO composite as a filler material in Matrimid^®^ 5218 (Figure 9a and Table 6), which was attributed to the improvement in polymer-filler interfacial adhesion brought by compositing the two fillers together. In contrast, physical blending UiO-66 and GO into the polymer matrix failed to achieve the same enhancements, as the individual fillers were unable to provide the same effective interfacial adhesion as that of the composite counterpart.

To ensure the success of the physical blending strategy, careful pairing of the fillers is important. For example, Valero et al. leveraged two different porous filler materials—an ordered mesoporous silica, MCM-41, and a MOF, MIL-53(Al)-NH_2_—in PSF matrix to improve filler dispersion and polymer-filler interfacial interaction [147]. They found that at 8 wt.% loading of each filler, MCM-41 was able to reduce MOF agglomeration, leading to tiny MIL-53(Al)-NH_2_ particles surrounding the shell of the silica spheres. As a result, H_2_/CH_4_ selectivity of the MMM was enhanced by 14%, reaching 67.3, while improving the H_2_ permeability by 67% (Table 6) [147]. The enhancements were attributed to the synergistic effect brought by the mesoporosity of MCM-41 as well as the microporosity and chemical compatibility of MIL-53(Al)-NH_2_ to the polymer matrix. Another attractive approach to achieving synergistic enhancements is to blend a 2D material to complement a 3D filler. Galve et al. used a JDF-L1 layered titanosilicate to pair with calcined MCM-41 3D spheres in a 6FDA-based copolyimide matrix for MMM preparation [148]. Owing to the strong barrier effect of the layered JDF-L1, H_2_/CH_4_ selectivity was able to enhance by 88%. At an optimal loading of 8 wt.% MCM-41 and 4 wt.% JDF-1, the H_2_ permeability was able to reach 440 barrer at H_2_/CH_4_ selectivity of 32.0, which corresponded to a 41% and 68% enhancement in permeability and selectivity, respectively (Table 6) [148].

## 5. Critical Evaluation of Hydrogen Separation Performances

On the basis of the discussions made in Section 4, we have summarized the H_2_ separation performances of representative MMMs in the recent literature (Table 2, Table 3, Table 4, Table 5 and Table 6). Here, a direct comparison of the separation performances across MMMs of different filler categories is hardly possible, given that the testing conditions, such as applied pressure, temperature, filler loading, and the use between single- and mixed-gas evaluations, are all not comparable. Nevertheless, certain trends are in general unambiguous. First, MOFs are seemingly the most common choice of filler, owing to their microporosity, and versatile chemical functionality that enables strong interfacial adhesion to the polymer matrix. Second, broadly speaking, 2D filler materials such as graphene-based materials and COFs tend to produce higher membrane selectivity, as a result of their high-aspect-ratios, leading to gas diffusion undertaking a more tortuous pathway through the polymer matrix. Third, although the binary filler strategy boasts synergistic enhancements in both permeability and selectivity, its true value lies more in minimizing loss in membrane selectivity, either through creating better polymer-filler interfacial interaction or the use of 2D materials to extend the transport pathway. As such, most MMMs with binary fillers show moderate to high membrane selectivity as compared to single-filler MMMs (Table 6). Here, we want to emphasize that the undesirable incompatibility between filler and polymer (e.g., sieve-in-a-cage, rigidified interface and plugged sieves) remains one of the Achille’s heels of MMMs, which can deteriorate membrane performances and cause the MMM strategy to backfire [44]. Hence, it is important to consider the psychochemical properties when choosing filler materials and fine-tune them for unlocking the true potential of MMMs. Fourth, most of the studies in Table 2, Table 3, Table 4, Table 5 and Table 6 did not report the permeation area of their membranes, and for those who did, the areas were typically in the scale of a few to tens of centimeters. There are clearly little efforts thus far to demonstrate the potential of scaling-up MMMs (see our discussion on scalability in Section 6). Last but not least, it is evident from the data that the intrinsic separation performance of the polymer matrix plays an active role in determining the performance outcome of the MMMs. For example, using an intrinsically high-permeable 6FDA-based polymer will ensure that the H_2_ permeability is high to start with, and hard to outperform by MMMs using low-permeable PSF and PI matrices, albeit permeability enhancements by the filler materials (see binary filler of Table 6). Hence, it is important to have a deep understanding of the intrinsic performance of polymer matrices, to allow rational pairing of filler materials with positive enhancements that complement the polymer matrices, for surpassing the H_2_ Robeson upper bounds (see Figure 3).

## 6. Conclusions and Perspectives

To conclude, we presented an overview of the current hydrogen market, focusing especially on existing technologies for hydrogen generation and purification, and how membrane separation can add value and contribute toward the future hydrogen economy. However, delivering the promise of membrane separation necessitates the evaluation of the current performance of polymeric membranes. Hence, in this review, we featured MMMs as one of the most compelling strategies to date, and singled out emerging filler materials, including zeolites, MOFs, COFs, and graphene-based materials, which we believe could change the game for MMMs. Further discussions on these fillers were first conducted, correlating the effects of physicochemical properties, such as porosity, morphology, and chemistry, on the polymer-filler interfacial interaction, and the ensuing H_2_ separation performances of the MMMs. Then, we examined the binary filler strategy and gained perspective on how two filler materials can create synergistic permeability-selectivity enhancements. A critical evaluation on the H_2_ separation performance data also provided key lessons learned for readers to take away from representative MMMs from these filler categories.

H_2_ membrane separation is in general challenging due to the fast diffusivity and low solubility of H_2_ molecules, leading to poor discrimination toward other penetrant gas molecules. Hence, moving forward, we argue that future development should focus on molecular-level tailoring of filler materials through fine-tuning porosity for increasing the molecular sieving capacity. This feat can be achieved by pre-synthetic engineering of the ligands or the strut length of the monomers used for MOF and COF syntheses, respectively. Alternatively, post-synthetic chemical functionalization of the pores can be used to create more constricted and well-defined pore sizes to target specifically H_2_ separation. On the same note, H_2_ molecules having fast diffusivity also demand a tighter polymer-filler interfacial morphology to mitigate defective MMMs. This therefore calls for optimized filler loadings, better dispersibility to prevent excessive agglomeration and greater interfacial compatibility through customizing the surface chemistry.

In addition, H_2_ separation under industrial relevant conditions typically involve high temperature as well as pressure. For example, the syngas produced by WGS reaction, following SMR, can have a temperature as high as above 200 °C [33], and stay at a pressure of at least 5–10 bar and beyond [150]. Hence, we suppose that future work on MMMs for H_2_ separation should place a stronger emphasis on membrane performances under elevated temperature and pressure. This includes demonstrating the thermal stability of the filler materials and the polymer matrices as well as understanding the optimal filler loadings to render robust mechanical properties of the MMMs to handle the applied pressure by the feed gas. As shown in Table 2, Table 3, Table 4, Table 5 and Table 6, there is currently scarce literature on H_2_ separation by MMMs at elevated temperatures, partly due to safety concerns and the availability of only a handful of polymeric materials with high glass transition temperatures. Moreover, there is a need to demonstrate chemical stability of filler materials and MMMs toward detrimental impurities in feed gases such as water, acidic gases and sulfur containing species [33]. Thus, taken together, these gaps need to be better addressed to provide a more accurate evaluation of MMMs in overcoming the permeability-selectivity tradeoff of polymeric membranes.

In terms of pairing filler materials to polymer matrices, there is a growing interest from the membrane community to see rational pairing for more meaningful designs of MMMs. As previously discussed, rational pairing involves the exploitation of specific attributes of filler materials to complement the limitations of polymer matrices (see Section 5). For example, Thür et al. recently found that the CO_2_ adsorption enthalpy (*Q*_st_) of MOF-808 gave a stronger correlation to the performance of MMMs as opposed to its intrinsic CO_2_ uptake, suggesting a possibility of thoughtful design of MOF-based MMMs by leveraging proven structural-performance indicators [151]. Furthermore, considering that the polymer matrices have a strong influence over the MMM performance (see discussion in Section 5), our group recently attempted to decouple the polymer matrix effect, and proposed a filler enhancement index (*F_index_*) that illuminates the true effectiveness of the filler by considering both permeability and selectivity enhancements for CO_2_/CH_4_ separation [44]. This index allows filler materials to be rated using a single composite metric for easy screening of polymer matrices to find the best match for pairing. In this age in which data represents a competitive edge, making data-driven decisions using these approaches is highly appealing and serves to meet the needs of rationally-designed MMMs for H_2_ separation.

Finally, demonstrating the scalability of MMMs is yet another milestone that demands a stronger commitment from both the industry and academia. Current research on MMMs for H_2_ separation are geared toward performance enhancements, without placing considerable effort into scaling-up MMMs to a scale that is practical for pilot-testing or test-bedding, as evidenced by the lack of information or small permeation areas as highlighted in Table 2, Table 3, Table 4, Table 5 and Table 6. Conversely, without crucial data such as this, there is limited buy-in from the industry, leaving MMMs a slow-to-adopt technology despite decades of academic research. Hence, for the academia, we call for greater efforts in translational research, such as scaling-up of MMMs for pilot-scale H_2_ separation evaluations as well as test-bedding, to showcase higher technology readiness level (TRL). Life-cycle assessments and techno-economic reviews should also be undertaken to provide more holistic assessments of the potential of MMMs for H_2_ separation. As for the industry, it is important to work collectively with academia by introducing more ground for exploration, feedback and co-creation such that academic research can stay relevant to the needs of the H_2_ market. We believe that it is only through collective and collaborative efforts such as these that the gaps for MMMs can be closed and technology transfer from laboratories to the market be successfully administered. With the impending hydrogen economy, demonstrating higher TRL and lowering the barrier for rationally-designed MMMs to reach commercialization will unquestionably strengthen the competitive position of membrane technology for H_2_ separation.

## Figures and Tables

**Table 1:** Hydrogen from different generation technologies and its common impurities [33].

Hydrogen Generation Technology	Common Impurity
Steam methane reforming	CO, CO_2_ and CH_4_
Partial oxidation of hydrocarbons	CO, CO_2_ and CH_4_
Gasification (coal/oil/biomass)	Light hydrocarbons, CO, CO_2_, CH_4_, O_2_, and N_2_
Electrolysis of water	CH_4_, O_2_, N_2_, CO_2_, and CO

**Table 2:** Summary of representative performances for zeolite-based membranes in H_2_-based separation.

Membrane		Separation Performance						Ref.
Filler	Polymer/Support	Test Condition	Permeation Area (cm^2^)	P(H_2_) (GPU)	Selectivity			
					H_2_/CO_2_	H_2_/CH_4_	H_2_/N_2_	
IL/SAPO-34 (1:4)	Pebax^®^ MH1657/PEGDME with ceramic	1 bar, 20 °C	-	4.9 (+188%)	0.11 (+31%)	1.2 (+9%)	-	[67]
IL/SAPO-34-NH_2_ (1:4)	Pebax^®^ MH1657/PEGDME with ceramic	1 bar, 20 °C	-	2.2 (+29%)	0.05 (−17%)	1.9 (+73%)	-	[67]
IL/SAPO-34-NH_2_ (1:2)	Pebax^®^ MH1657/PEGDME with ceramic	1 bar, 20 °C	-	2.3 (+35%)	0.05 (−17%)	2.6 (+136%)	-	[67]
SAPO-34	Pebax^®^ MH1657/PEGDME with ceramic	1 bar, 20 °C	-	1.5 (−12%)	0.06 (0%)	1.4 (+27%)	-	[67]
DD3R (20 wt%)	Matrimid^®^ 5218	1 bar, 25 °C	11.95	34.9 ^a^ (+105%)	-	375 (+188%)	-	[68]
Zeolite 4A (25 wt%)	PVAc	-	2.3–2.5	3.8 ^a^ (−36%)	1.6 (−20%)	-	156 (+42%)	[69]
Zeolite 4A (15 wt%)	PVAc	0.75 bar, 30 °C	-	5.8 ^a^ (0%)	5.3 (+13%)	-	117 (+22%)	[71]
Modified zeolite 4A (15 wt%)	PVAc	0.75 bar, 30 °C	-	5.6 ^a^ (−3%)	6.1 (+30%)	-	143 (+49%)	[71]
Hydroxyl sodalite (5 wt%)	PSF	-	-	21.8 (+169%)	1.1 (−78%)	-	1.1 (−78%)	[72]
Silica sodalite (15 wt%)	PSF	-	-	22.8 (+182%)	1.1 (−78%)	-	1.0 (−80%)	[72]
HZS (8 wt%)	6FDA-DAM	2 bar, 35 °C	28	541 ^a^ (+13%)	0.77 (−3%)	25 (+47%)	21 (+62%)	[78]

^a^ Permeability reported in the units of barrer; numbers in the parentheses represent the percentage enhancements with respect to the pristine polymeric membranes. 6FDA, 4,4′(hexafluoroisopropylidene)diphthalic anhydride; DAM, 2,4,6-triphenyl-m-phenylenediamine; PEGDME, poly(ethylene glycol) dimethyl ether; PSF, polysulfone; PVAc, polyvinyl acetate.

**Table 3:** Summary of representative performances for MOF-based membranes in H_2_-based separation.

Membrane		Separation Performance						Ref.
Filler	Polymer/Support	Test Condition	Permeation Area (cm^2^)	P(H_2_) (GPU)	Selectivity			
					H_2_/CO_2_	H_2_/CH_4_	H_2_/N_2_	
CAU-1-NH_2_ (15 wt%)	PMMA	3 bar, 25 °C	0.02	11,000 ^a^ (+122%)	13 (+333%)	-	-	[84]
CBMN (2 wt%)	6FDA-Durene-DABA	3 bar, 25 °C	-	410 ^a^ (−24%)	1.2 (−14%)	29 (+45%)	42 (+83%)	[113]
[Cu_2_(ndc)_2_(dabco)]_n_ ns (30 wt%)	PBI	2 bar, 35 °C	-	12.1 ^a^ (+236%)	11 (+21%)	-	-	[104]
MIL-53(Al)-NH_2_ (20 wt%)	PI-1388 (VTEC^TM^)	5 bar, 35 °C	16	5.4 ^a^ (+8%)	5.4 (+8%)	-	-	[86]
NH_2_-CAU-1 (20 wt%)	PMMA with ceramic	2 bar	-	92 (+475%)	33 (+400%)	-	-	[87]
NH_2_-MIL-53 (20 wt%)	PMMA with ceramic	2 bar	-	72 (+350%)	45 (+582%)	-	-	[87]
nZIF-7	PEI	2 bar, 35 °C	-	1.1 (−45%)	3.2 (−68%)	42 (+324%)	54 (+38%)	[95]
PSM-nZIF-7	PEI	2 bar, 35 °C	-	8.6 (+330%)	9.9 (−1%)	23 (+132%)	13 (−67%)	[95]
UiO-66 (3 wt%)	PAA/PVP	-	-	1.2 (−56%)	20.3 (+62%)	-	-	[98]
UiO-66-NH_2_ (55 wt%)	6FDA-DAM:DABA (3:2)	3 bar, 35 °C	-	2932 ^a^ (+1529%)	1.2 (+20%)	34 (+3%)	24 (+4%)	[89]
UiO-66-NH_2_ (40 wt%)	6FDA-DAM	3 bar, 35 °C	-	1810 ^a^ (+165%)	0.7 (−13%)	10 (−29%)	10 (−23%)	[89]
UiO-66-NH_2_ (40 wt%)	6FDA-BPDA-DAM (1:1)	3 bar, 35 °C	-	1086 ^a^ (+198%)	0.8 (+14%)	12 (+14%)	13 (+13%)	[89]
UiO-66-NH_2_ (18 wt%)	PVP/PEI	1 bar, 25 °C	-	31 ^a^ (+244%)	0.08 (−81%)	-	-	[88]
UiO-66(Hf)-(OH)_2_ (10 wt%)	PBI	2 bar, 35 °C	-	8.2 ^a^ (+128%)	12 (+33%)	-	-	[82]
Oriented & Penetrating ZIF-7	PI	2 bar, 100 °C	-	889 (+1357%) ^b^	92 (+889%) ^b^	-	128 (+1424%) ^b^	[85]
ZIF-7-I	6FDA-DAM with α-alumina	-	-	921 ^a^ (+56%)	2 (+100%)	67 (+68%)	36 (+16%)	[99]
ZIF-7-III	6FDA-DAM with α-alumina	-	-	322 ^a^ (−45%)	4 (+300%)	172 (+330%)	59 (+90%)	[99]
ZIF-7-mix	6FDA-DAM with α-alumina	-	-	478 ^a^ (−45%)	4 (+300%)	86 (+115%)	32 (+3%)	[99]
ZIF-8 (5 wt%)	PSF	4 bar, 30 °C	-	53 ^a^ (+43%)	2.3 (+5%)	57 (+24%)	58 (+21%)	[100]
ZIF-8 (20 wt%)	6FDA-Durene	1 bar, 35 °C	-	1525 ^a^ (+80%)	1 (+11%)	16 (+11%)	14 (+18%)	[102]
ZIF-8-PD (20 wt%)	6FDA-Durene	1 bar, 35 °C	-	1320 ^a^ (+56%)	1 (+11%)	16 (+11%)	17 (0%)	[102]
ZIF-8 (40 wt%)	Matrimid^®^ 5218	3.5 bar, 35 °C	-	400 ^a^ (+1329%)	4 (+33%)	50 (−52%)	44 (−50%)	[103]
ZIF-8-DA (40 wt%)	Matrimid^®^ 5218	3.5 bar, 35 °C	-	65 ^a^ (+132%)	4 (+33%)	108 (+4%)	130 (+48%)	[103]
ZIF-8 (20 wt%)	TR polymers (90% conversion)	1 bar, 35 °C	3.14	1206 ^a^ (+189%)	1.3 (+30%)	28 (+22%)	21 (0%)	[97]
ZIF-93 (20 wt%)	PBI	3 bar, 180 °C	3.14	128 ^a^ (+184%)	5 (+25%)	-	-	[96]
ZIF-L (20 wt%)	PI	1 bar	-	260 ^a^ (+18%)	13 (+550%)	62 (−11%)	41 (+3%)	[111]
ZIF-L-Co (20 wt%)	TB	2 bar, 24 °C	-	1236 ^a^ (+312%)	2 (0%)	26 (+48%)	25 (−49%)	[112]
ZIF-L-Zn (20 wt%)	TB	2 bar, 24 °C	-	898 ^a^ (+199%)	2 (0%)	25 (+50%)	23 (−53%)	[112]

^a^ Permeability reported in the units of barrer; ^b^ percentage enhancement calculated with respect to the conventional unoriented MMM. Numbers in the parentheses represent the percentage enhancements with respect to the pristine polymeric membranes; 6FDA, 4,4′(hexafluoroisopropylidene)diphthalic anhydride; CBMN, co-benzenedicarboxylate MOF nanosheet; DAM, 2,4,6-triphenyl-*m*-phenylenediamine; DABA, 3,5-diaminobenzoic acid; ndc, 1,4-naphthalene dicarboxylate; dabco, 1,4-diazabicyclo(2.2.2)octane; ns, nanosheets; PAA, poly(acrylic acid); PBI, polybenzimidazole; PD, polydopamine; PEI, polyethyleneimine; PI, polyimide; PMMA, poly(methyl methacrylate); PPO, poly(phenylene oxide); PVP, polyvinylpyrrolidone; TB, Troger’s base; TPIM, phenazine-containing triptycene ladder polymers; TR, thermally rearranged.

**Table 4:** Summary of representative performances for COF-based membranes in H_2_-based separation.

Membrane		Separation Performance						Ref.
Filler	Polymer/Support	Test Condition	Permeation Area (cm^2^)	P(H_2_) (GPU)	Selectivity			
					H_2_/CO_2_	H_2_/CH_4_	H_2_/N_2_	
COF_L_	PVAm/mPSF	5 bar	-	90 (+221%)	0.1 (0%)	-	-	[131]
COF_M_	PVAm/mPSF	5 bar	-	85 (+204%)	0.085 (−15%)	-	-	[131]
COF_P_	PVAm/mPSF	5 bar	-	58 (+107%)	0.052 (−48%)	-	-	[131]
PAOPIM-1 (10 wt%)	PI-COOH	3 bar	-	1279 ^a^ (+3%)	1 (+11%)	16.7 (+14%)	23.5 (+22%)	[132]
NUS-2 (20 wt%)	Ultem	2 bar, 35 °C	-	22.7 ^a^ (+255%)	4.6 (+59%)	103 (+78%)	-	[130]
NUS-2 (20 wt%)	PBI	5 bar, 35 °C		4.1 ^a^ (+14%)	31.4 (+231%)	-	-	[130]
NUS-3 (20 wt%)	Ultem	2 bar, 35 °C	-	33.4 ^a^ (+423%)	2.2 (−24%)	63 (+9%)	-	[130]
NUS-3 (20 wt%)	PBI	5 bar, 35 °C		12.2 ^a^ (+239%)	8.9 (−6%)	-	-	[130]
*p*-DCX (10 wt%)	PTMSP	2 bar, 25 °C	-	30,000 ^a^ (+53%)	0.7 (+17%)	2.8 (0%)	5.0 (+4%)	[125]
V-125 (10 wt%)	PTMSP	2 bar, 25 °C	-	11,100 ^a^ (−43%)	0.7 (+17%)	4.3 (+54%)	7.6 (+62%)	[125]
PAF-1 (10 wt%)	PTMSP	2 bar, 25 °C	-	18,400 ^a^ (−6%)	0.7 (+17%)	4.8 (+72%)	8.3 (+77%)	[125]
PAF-1 (10 wt%)	TPIM-2	-	-	4886 ^a^ (+196%)	1 (+25%)	18.8 (+18%)	23.4 (+20%)	[126]
PAF-1 (10 wt%)	PIM-1	-	-	5500 ^a^ (+375%)	-	-	4.5 (+13%)	[127]

^a^ Permeability reported in the units of barrer; numbers in the parentheses represent the percentage enhancements with respect to the pristine polymeric membranes; PI, polyimide; PIM-1, polymer of intrinsic microporosity; PSF, polysulfone; PVAm, polyvinylamine; TPIM, phenazine-containing triptycene ladder polymers.

**Table 5:** Summary of representative performances for graphene-based membranes in H_2_-based separation.

Membrane		Separation Performance						Ref.
Filler	Polymer/Support	Test Condition	Permeation Area (cm^2^)	P(H_2_) (GPU)	Selectivity			
					H_2_/CO_2_	H_2_/CH_4_	H_2_/N_2_	
CGO-76 (C=Cysteamine)	Anodized Al_2_O_3_	1.5 bar, 25 °C	3.14	52 (−45%) ^b^	21 (+133%) ^b^	-	-	[143]
GO (8 wt%)	PSF	35 °C	-	4.7 ^a^ (−61%)	-	29 (−51%)	-	[135]
GO (8 wt%)	PI	35 °C	-	14 ^a^ (−55%)	-	82 (−39%)	-	[135]
GO/PEI	Porous Al_2_O_3_	25 °C	3.14	1000 ^a^ (−83%)	29 (+625%)	-	-	[139]
GO/EDA-2	Porous Al_2_O_3_	25 °C	3.14	73 (−42%) ^b^	23 (+35%)	-	-	[140]

^a^ Permeability reported in the units of barrer; ^b^ percentage enhancement calculated with respect to pristine GO-laminated membranes; numbers in the parentheses represent the percentage enhancements with respect to the pristine polymeric membranes. PEI, polyethyleneimine; EDA-2, 2 h pre-crosslinking with ethylenediamine

**Table 6:** Summary of representative performances for MMMs with binary fillers in H_2_-based separation.

Membrane		Separation Performance						Ref.
Filler	Polymer/Support	Test Condition	Permeation Area (cm^2^)	P(H_2_) (GPU)	Selectivity			
					H_2_/CO_2_	H_2_/CH_4_	H_2_/N_2_	
UiO-66 + GO (8 wt%)	PSF	35 °C	-	12 ^a^ (0%)	-	60 (+2%)	-	[135]
UiO-66 + GO (8 wt%)	PI	35 °C	-	41 ^a^ (+32%)	-	136 (+1%)	-	[135]
MCM-41 + MIL-53(Al)-NH_2_ (8 wt%)	PSF	35 °C	15.2	20 ^a^ (+67%)	-	67 (+14%)	-	[147]
MCM-41 + MIL-53(Al)-NH_2_ (8 wt%)	PI	35 °C	15.2	16 ^a^ (−47%)	-	132 (0%)	-	[147]
MCM-41 + JDF-L1 (8 + 4 wt%)	6FDA-based copolyimide	35 °C	-	440 ^a^ (+41%)	-	32 (+68%)	-	[148]
HKUST-1 + Silicalite-1 (8 wt%)	PSF	35 °C	15.2	16 ^a^ (+33%)	-	83 (+38%)	-	[149]
ZIF-8 + Silicalite-1 (8 wt%)	PSF	35 °C	15.2	17 ^a^ (+42%)	-	74 (+23%)	-	[149]

^a^ Permeability reported in the units of barrer; numbers in the parentheses represent the percentage enhancements with respect to the pristine polymeric membranes; 6FDA, 4,4′(hexafluoroisopropylidene)diphthalic anhydride; PI, polyimide; PSF, polysulfone.

## Data Availability

Not applicable.
